# Expression Analysis of the Ligands for the Natural Killer Cell Receptors NKp30 and NKp44

**DOI:** 10.1371/journal.pone.0001339

**Published:** 2007-12-19

**Authors:** Andreina Byrd, Sabrina C. Hoffmann, Mostafa Jarahian, Frank Momburg, Carsten Watzl

**Affiliations:** 1 Institute for Immunology, University Heidelberg, Heidelberg, Germany; 2 Department of Molecular Immunology, German Cancer Research Center, Heidelberg, Germany; Massachusetts General Hospital and Harvard Medical School, United States of America

## Abstract

**Background:**

The natural cytotoxicity receptors (NCR) are important to stimulate the activity of Natural Killer (NK) cells against transformed cells. Identification of NCR ligands and their level of expression on normal and neoplastic cells has important implications for the rational design of immunotherapy strategies for cancer.

**Methodology/Principal Findings:**

Here we analyze the expression of NKp30 ligand and NKp44 ligand on 30 transformed or non-transformed cell lines of different origin. We find intracellular and surface expression of these two ligands on almost all cell lines tested. Expression of NKp30 and NKp44 ligands was variable and did not correlate with the origin of the cell line. Expression of NKp30 and NKp44 ligand correlated with NKp30 and NKp44-mediated NK cell lysis of tumor cells, respectively. The surface expression of NKp30 ligand and NKp44 ligand was sensitive to trypsin treatment and was reduced in cells arrested in G_2_/M phase.

**Conclusion/Significance:**

These data demonstrate the ubiquitous expression of the ligands for NKp30 and NKp44 and give an important insight into the regulation of these ligands.

## Introduction

Natural Killer (NK) cells represent a unique subset of lymphocytes that can mediate innate immune responses against tumors and certain pathogens [1]–[3]. Many studies have demonstrated the effectiveness of NK cells to attack tumor cell lines or freshly isolated primary tumor cells *in vitro* [4]. In murine models, NK cell activity is important in the immune reaction against transplanted or induced tumors *in vivo* [5], [6]. In humans, recent studies with patients receiving hematopoietic stem cell transplantation demonstrated an important role of NK cells for eradicating certain types of hematopoietic tumors [7]. This potent anti-tumor activity of NK cells is regulated by the engagement of NK cell surface receptors, cytokines and the crosstalk with other immune cells [8]–[10]. NK cell receptors can roughly be divided into inhibitory and activating receptors. Many inhibitory receptors recognize certain MHC class I alleles and thereby ensure the tolerance of NK cells against self [11]. However, also non-MHC class I recognizing inhibitory receptors seem to be important for regulating NK cell activity [12]. Activating receptors on human NK cells include CD16, NKG2D, the natural cytotoxicity receptors (NCR) NKp30, NKp44 and NKp46, DNAM-1, 2B4 (CD244), NTB-A, CRACC (CS1) and NKp80 [13]. In recent years, most of the ligands recognized by these receptors have been identified.

The NCR have been shown to be important receptors for the stimulation of anti-tumor activity of human NK cells [3]. Anti-NCR monoclonal antibodies can block NK cell-mediated lysis of many different tumor cell lines [14]–[17] and the NK cell-mediated surveillance of mitotic cells [18]. NKp30 is also involved in the crosstalk between NK cells and dendritic cells [19]. Furthermore, the expression of NCR on NK cell clones correlates with their ability to lyse tumor cells [17]. This indirectly demonstrates that ligands for NCR are expressed by many transformed, but also by normal cells. However, the identity of the NCR ligands is still unknown to date. Pathogen-derived structures have been shown to interact with NCR. The hemagglutinin protein of several viruses can bind and activate NKp46 and NKp44 [20], [21]. The pp65 protein of HCMV has been shown to bind NKp30 and inhibit its function [22]. The Plasmodium falciparum erythrocyte membrane protein-1 is involved in the NCR-mediated NK cell attack against infected erythrocytes [23]. Pathogens such as vaccinia virus, HIV or herpes simplex virus have also been shown to up-regulate the expression of cellular NCR ligands in infected cells [24]–[26]. Heparan sulfate structures have been postulated as cellular ligands for NCR but functional consequences for these interactions are debated [27]–[29].

A direct probe for the expression of NCR ligands is the use of soluble NCR fusion proteins as staining reagents. Such approaches have shown the existence of NCR ligands on several tumor cell lines [30], [31]. Here we perform an analysis of the expression of NKp30 ligand (NKp30L) and NKp44 ligand (NKp44L) on a panel of 30 cell lines of different origin. We demonstrate that NKp30L and NKp44L are ubiquitously and variably expressed on transformed and non-transformed cell lines. The surface expression of NKp30L and NKp44L correlates with sensitivity towards NK cell lysis, is sensitive to trypsin treatment and is down-modulated in cells arrested in G_2_/M phase.

## Results

**Figure 1:**
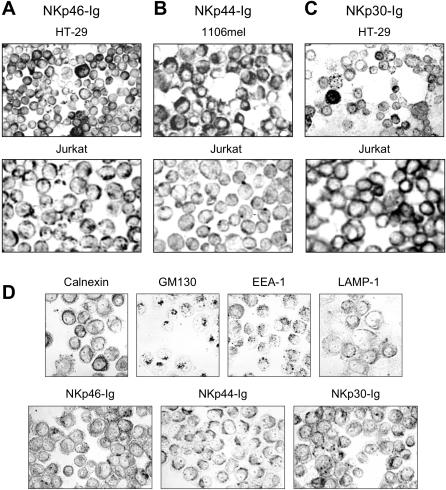
Immunoperoxidase stainings of acetone-fixed tumor cells on cytospins. Intracellular compartments of HT-29, 1106mel, Jurkat (A–C) and HeLa cells (D) were stained with NKp46-Ig, NKp44-Ig and NKp30-Ig fusion proteins as indicated. In (D), intracellular stainings of HeLa cells with the ER marker calnexin, the Golgi marker GM130, the early endosome marker EEA-1, and the late endosome marker LAMP-1 were performed in parallel for comparison with NCR-Ig staining pattern. A–C, ×150, D, ×250.

For the intracellular detection of NCR ligands, we initially made use of NCR-Ig fusion proteins. With these reagents we stained 11 tumor cell lines of different origin (Table 1). We obtained specific staining of intracellular structures in all cell lines tested. As representative examples, HT-29, CHO, Jurkat and HeLa cells are shown in Fig. 1. Interestingly, the staining pattern of the three different NCR-Ig fusion proteins was specific and reproducible in the different cell lines tested. The comparison with parallel cytospin stainings using antibodies against the ER marker calnexin, the Golgi marker GM130, the endosomal marker EEA-1, and the late endosomal marker LAMP-1, respectively, revealed i) that NKp46-Ig labels the ER and a subset of early endosomes of HeLa cells, ii) that NKp44-Ig appeared to label late endosomes in a very homogenous fashion, and iii) that NKp30-Ig weakly stained the ER and strongly labeled a heterogenous population of large peripheral vesicles which probably represent early endosomal compartments (Fig. 1D). These findings suggest that recognition structures for the three NCR are readily detectable in all cells tested.

**Table 1:** 

Cell line	Description
293T	Embryonic kidney cell line
A125	Lung adenocarcinoma cell line
A549	Lung adenocarcinoma cell line
1106mel	Melanoma cell line
ZKR	Melanoma cell line
HeLa	Cervix carcinoma cell line
HT-29	Colon carcinoma cell line
LoVo	Colon carcinoma cell line
BJAB	B-cell lymphoma
Jurkat	T-cell lymphoma
CHO	Hamster ovarian cell line

**Figure 2:**
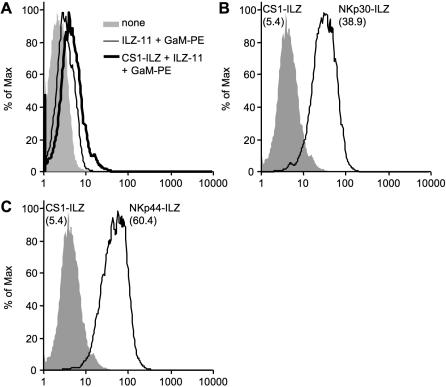
Detection of NKp30L and NKp44L on the surface of cells by FACS analysis. (A) 293T cells were analyzed without treatment (none) or after incubation with medium or CS1-ILZ, followed by anti-ILZ mAb (ILZ-11) and PE-labeled goat anti-mouse IgG (GaM-PE) staining. (B, C) 293T cells were stained with CS1-ILZ, NKp30-ILZ (B) or NKp44-ILZ (C) as described in (A) and analyzed by FACS. Mean fluorescence intensities of the stainings are indicated in brackets.

**Figure 3:**
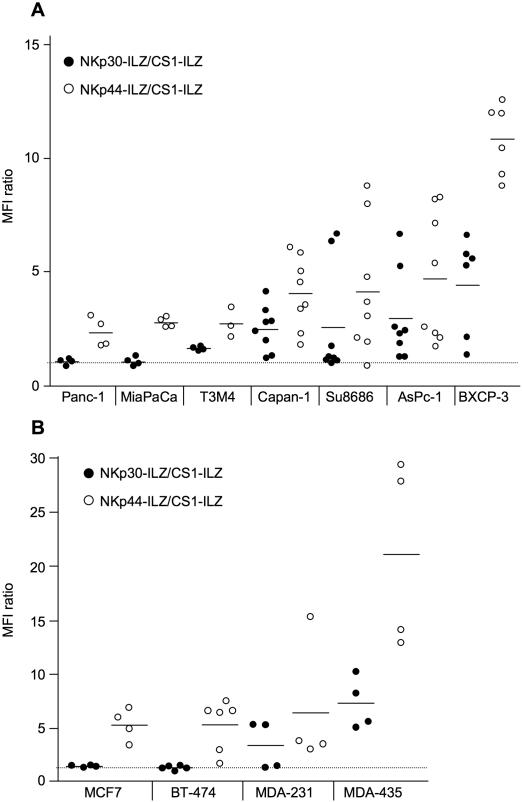
Analysis of NKp30L and NKp44L expression on pancreatic carcinoma cell lines (A) and breast carcinoma cell lines (B). The indicated cells were stained with CS1-ILZ, NKp30-ILZ or NKp44-ILZ and analyzed by FACS. The mean fluorescence intensities (MFI) of the NKp30-ILZ (black circles) and NKp44-ILZ (white circles) stainings were divided by the MFI of CS1-ILZ background staining. Each dot represents the result of an independent staining.

To directly test for the presence of NCR ligands on the surface of cells, we used the extracellular domain of NKp30, NKp44 and NKp46 and fused it to an aminoterminal isoleucin-zipper (ILZ) sequence, which induces trimerization [32]. In contrast to Ig fusion proteins, the ILZ fusion proteins show higher avidity for surface staining and do not bind to Fc receptors [32]. The NCR-ILZ fusion proteins were produced in the human cell line 293T, purified and used for the staining of 30 cell lines of different origin (Table 2). Binding of the NCR-ILZ was detected by a monoclonal anti-ILZ antibody and analyzed by FACS. As a control, we used a CS1-ILZ fusion protein, which only showed background staining on the cells tested (Fig. 2 and data not shown). An example for the surface staining of NKp30-ILZ and NKp44-ILZ is shown in Fig. 2. We did not observe any significant staining of NKp46-ILZ. A possible explanation for this lack of activity could be that the ILZ part obscures the D2 domain of NKp46, which is important for ligand binding [30]. We therefore analyzed the expression of NKp30L and NKp44L on the different cell lines. To make the results between the different experiments comparable, we normalized all FACS results to the CS1-ILZ background staining. Most cell lines were tested between four and six times for the expression of NKp30L and NKp44L in independent experiments. First we analyzed seven tumor cell lines derived from pancreatic cancer (Fig. 3A). The expression of NKp30L and NKp44L was very variable, ranging from no detectable expression to significant surface expression. NKp44L staining was consistently higher than NKp30L. This could indicate, that the expression of NKp44L is higher than that of NKp30L. However, this could also be caused by better staining efficiency or higher affinity binding of the NKp44-ILZ. Interestingly, the staining intensities for NKp30L and NKp44L seemed to be linked with cells expressing high amounts of NKp30L also expressing high amounts of NKp44L. This variable but linked expression was also observed in four different breast carcinoma cell lines tested (Fig. 3B).

**Table 2:** 

Cell line	Description
Panc-1	Pancreatic cancer cell line
Capan-1	Pancreatic cancer cell line
Su8686	Pancreatic cancer cell line
BXCP-3	Pancreatic cancer cell line
MiaPaCa	Pancreatic cancer cell line
T3M4	Pancreatic cancer cell line
AsPc-1	Pancreatic cancer cell line
MCF7	Breast cancer cell line
BT-474	Breast cancer cell line
MDA-231	Breast cancer cell line
MDA-435	Breast cancer cell line
293T	Embryonic kidney cell line
A125	Lung adenocarcinoma cell line
A549	Lung adenocarcinoma cell line
1106mel	Melanoma cell line
HeLa	Cervix carcinoma cell line
Shep	Neuroblastoma cell line
Huh7	Hepatoma cell line
Colo201	Colon carcinoma cell line
Daudi	Burkitt lymphoma cell line
Raji	Burkitt lymphoma cell line
Namalwa	Burkitt lymphoma cell line
K562	Chronic myeloid leukemia (CML) cell line
EM-3	Chronic myeloid leukemia (CML) cell line
THP-1	Acute monocytic leukemia (AML) cell line
HL-60	Acute monocytic leukemia (AML) cell line
721.221	EBV immortalized B cell line
SD-1	EBV immortalized acute lymphocytic leukemia (ALL) cell line
HFF	Primary human foreskin fibroblasts
Osteoblasts	Primary murine osteoblasts

**Figure 4:**
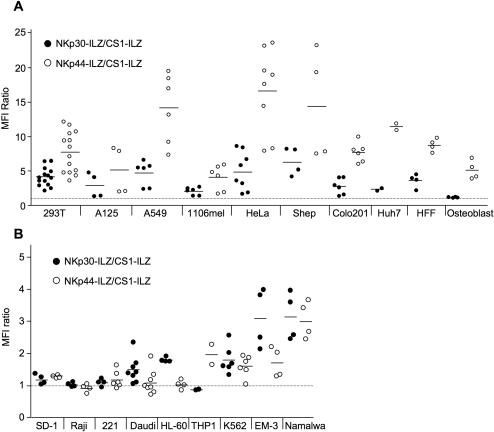
Analysis of NKp30L and NKp44L expression on solid tumor cell lines of different origin (A) and of cell lines hematopoietic origin (B). The indicated cells were analyzed for NKp30L and NKp44L expression as described in figure 3.

When we extended our analysis to more tumor cell lines of different origin we detected NKp30L and NKp44L on the surface of almost all cell lines tested (Fig. 4A). This indicates that the ligands for NKp30 and NKp44 are widely expressed on tumor cells, consistent with the finding that these NK cell receptors are involved in the lysis of many different tumors. However, not only transformed cells showed expression of NKp30L and NKp44L. We could also detect the presence of NKp30L and NKp44L on primary human foreskin fibroblasts and found NKp44L even on murine primary osteoblasts (Fig. 4A). NK cells are particularly effective in attacking hematological malignancies. Interestingly, when testing cell lines of hematological origin, we detected much less surface staining of NKp30L and NKp44L when compared to the solid tumors tested (Fig. 4B). Also, the staining of NKp30L and NKp44L was not linked in these tumor cell lines with HL-60, K562 and EM-3 showing higher NKp30L than NKp44L expression. Primary PBMCs did not show any staining for NKp30L and NKp44L (data not shown). This suggests that the NCR ligands are less expressed in cell lines of hematological origin.

**Figure 5:**
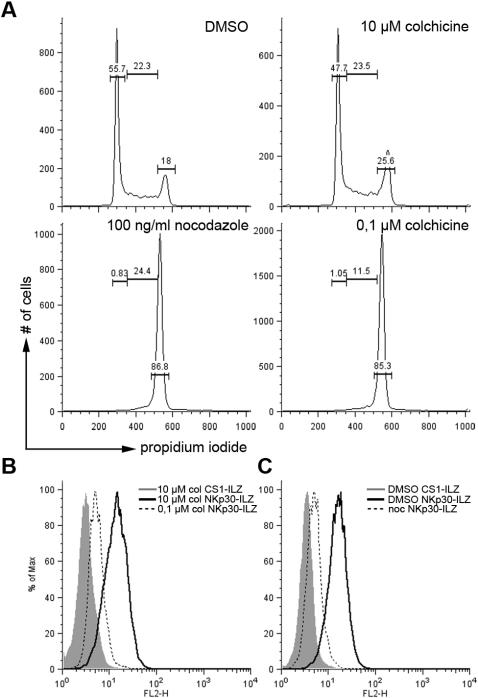
Reduced expression of NKp30L in cells arrested in mitosis. (A) 293T cells were treated with DMSO for 18 hours, 10 µM colchicine (col) for 1 hour, 100 ng/ml nocodazole (noc) or 0.1 µM colchicine for 18 hours and the cell cycle stage of the treated cells was analyzed by FACS. Numbers represent percentage of cells in G_1_ phase (left) S phase (middle) and G_2_/M phase (right). (B and C) the same cells as in (A) were analyzed for the expression of NKp30L as described in Fig. 2.

In some cases we observed relatively wide variations in NKp30L and NKp44L expression when repeatedly staining the same cell line (see MDA-435 in Fig. 3B or Shep in Fig. 4A). It was recently shown that NK cells have the ability to survey specifically cells in mitosis and that NCR are involved in this enhanced surveillance [18]. We therefore investigated if the expression of NKp30L was cell cycle regulated. We used colchicine or nocodazole to arrest cells in G_2_/M phase (Fig. 5A). As a control we used DMSO or high concentrations of colchicine for a short period of time to disrupt the microtubules without arresting cells in G_2_/M. While these cells or DMSO treated cells showed clearly detectable NKp30-ILZ staining, the expression of NKp30L was reduced in cells arrested in G_2_/M phase after colchicine (Fig. 5B) or nocodazole (Fig. 5C) treatment. Similar results were obtained for NKp44L expression and using Ig-fusion proteins (data not shown). This demonstrates that NKp30L and NKp44L are cell cycle-regulated, but that these ligands are actually down-regulated during mitosis.

**Figure 6:**
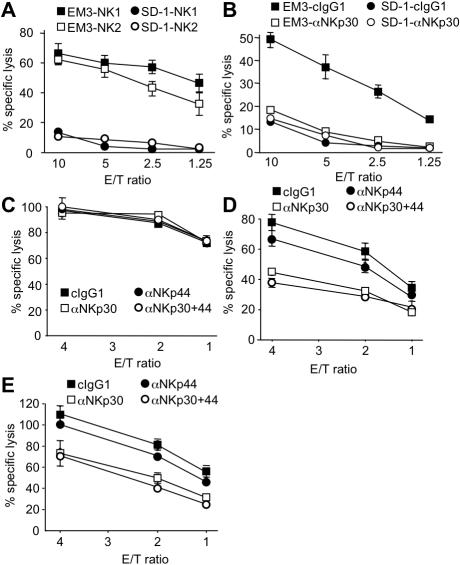
Correlation between NKp30L and NKp44L expression and NKp30 and NKp44-mediated lysis by NK cells. (A) EM-3 and SD-1 cells were used as targets in a 4 h ^51^Cr release assay using IL2-activated NK cells from two different donors (NK1 and NK2) as effector cells at the indicated effector to target (E/T) ratios. (B) ^51^Cr release assay of EM-3 and SD-1 target cells in the presence of control antibodies (cIgG1) or blocking anti-NKp30 antibodies (αNKp30). (C–E) Lysis of K562 (C), HeLa (D) and Shep (E) cells by IL-2 activated NK cells in the presence of control antibodies (cIgG1) or blocking anti-NKp30 (αNKp30) and/or anti-NKp44 antibodies (αNKp44).

To correlate the expression analysis with functional data, we first compared SD-1 cells showing no detectable NKp30L and NKp44L expression with EM-3 cells, which showed good expression of NKp30L and only weak expression of NKp44L (Fig. 4B). When comparing the lysis of these two cell lines by IL-2-activated primary NK cells from two different donors we detected good lysis of EM-3 but weak lysis of SD-1 target cells, correlating with the NCR ligand expression (Fig. 6A). More importantly, the lysis of EM-3 cells could be reduced to the level of SD-1 killing by using a blocking anti-NKp30 antibody, while this antibody did not reduce the lysis of SD-1 cells (Fig. 6B). This again correlates with the staining data for the expression of NKp30L on these two cell lines. To check if the expression level of NKp30L correlates with the NKp30-dependent lysis of tumor cells we tested K562 cells, which showed detectable, but lower NKp30L expression compared to EM-3 cells. Lysis of K562 cells could not be blocked by anti-NKp30 nor by anti-NKp44 antibodies (Fig. 6C). This demonstrates that a certain threshold of NKp30L expression is necessary to induce significant NKp30-dependent lysis. To check if the higher staining intensity for NKp44L on HeLa and Shep also correlates with NKp44-mediated lysis we tested the NK cell-mediated killing of these cells. Lysis of both cells could be blocked by anti-NKp30 and anti-NKp44 antibodies (Fig. 6D, E). However, the reduction mediated by blocking NKp30 was larger than the reduction mediated by NKp44 blockade. This is in contrast to the staining intensities in the FACS analysis (Fig. 4A).

**Figure 7:**
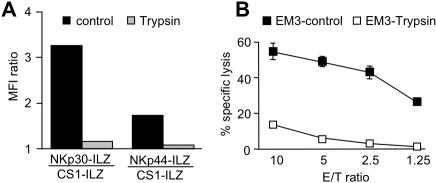
Surface expression of NKp30L and NKp44L is sensitive to Trypsin treatment. EM-3 cells were left untreated (control) or treated with trypsin. (A) The expression of NKp30L and NKp44L was then analyzed as described in figure 3 or cells were used as targets in a ^51^Cr release assay (B).

The nature of NKp30L and NKp44L is still unclear. Recent publications point towards an involvement of heparan sulfate structures in the binding of NKp30 and NKp44. However, we found that also protein structures are important for the binding of NCR, as trypsin abolished the binding of NKp30-ILZ and NKp44-ILZ to the surface of treated EM-3 cells (Fig. 7A). This reduction in surface expression of NKp30L and NKp44L after trypsin treatment also had functional consequences. Removal of NKp30L from the surface of EM-3 cells significantly reduced the lysis of these cells (Fig. 7B), which is largely dependent on the engagement of NKp30 (Fig. 6B).

## Discussion

NCR are important activating receptors for the anti-tumor activity of NK cells. However, only limited information exists about the nature of the NCR ligands. Direct analysis of the expression of NCR ligands has only been performed on a limited number of tumor cells [30], [31]. Here we provided a more comprehensive analysis of the expression of NKp30L and NKp44L. The surface expression of NKp46L could not be analyzed, as our NKp46-ILZ fusion protein did not give any significant surface staining on several cells reported to express NKp46L. The second Ig domain (D2) of NKp46 is essential for ligand recognition [30]. This domain is directly adjacent to the ILZ sequence in our fusion protein. One reason for the lack of NKp46-ILZ activity could be the steric hindrance of the D2 domain by the ILZ sequence.

For NKp30L and NKp44L we found intracellular and extracellular expression on almost all cell lines tested. Expression of these ligands was not restricted to cell lines of any specific origin. Instead, we found wide variations in NKp30L and NKp44L surface expression among cell lines of the same histogenetic origin. This is in agreement with an earlier study, which found variable expression of NCR ligands in melanoma cells [15]. Interestingly, we detected linked expression of NKp30L and NKp44L on many cell lines derived from solid tumors. This is unlikely to be an artifact or a cross-reactivity of our staining reagents as we did not find such linked expression on cells of hematopoietic origin. The different staining intensities of NKp30-ILZ and NKp44-ILZ could either reflect a different expression of NKp30L and NKp44L, or be simply the result of a higher affinity between NKp44L and the NKp44 receptor. However, in the absence of an identified cellular ligand for these receptors this questions cannot be answered. Functionally, the higher staining intensity foe NKp44L does not translate in higher NKp44-dependent lysis. To the contrary, NKp30-dependent lysis was even higher than NKp44-dependent lysis in the Shep and HeLa cell lines (Fig. 6). However, the staining intensity for each ligand is indicative for the lysis. EM3 and K562 cells, showing low staining intensity for NKp44L were not lysed in an NKp44-dependent fashion, whereas Shep and HeLa cells, showing high staining intensity for NKp44L were lysed in a NKp44-dependent way. Similarly, K562 cells, showing low NKp30L staining intensity were not lysed in an NKp30-dependent fashion, while EM-3, HeLa and Shep cells were. How much lysis of a given target cell depends on NKp30 or NKp44 will also depend on the expression of other activating NK cell ligands. Therefore, the amount of NKp30L or NKp44L staining intensity cannot predict the amount of NKp30 or NKp44-dependent NK cell lysis.

Heparan sulfate was reported to be involved in the recognition of NCR, suggesting the recognition of similar ligands by these receptors [27], [28]. However, the differential expression of NKp30L and NKp44L by the cell lines tested in this study suggests the existence of distinct ligands for both receptors. Also, the stainings of intracellular compartments with NKp46-Ig, NKp44-Ig and NKp30-Ig revealed clearly different patterns, strongly suggesting that these receptors recognize distinct target structures. The highly reproducible and strong vesicular, intracellular stainings as compared with the more variable surface stainings suggests that the surface expression of NCR ligands may be regulated by plasma membrane turnover. Changes in internalization rates might provide tumor cells with a means to escape from NK cell attack.

The NCR ligands are likely to contain a protein component as we found the surface expression of NKp30L and NKp44L to be sensitive to trypsin treatment. This has important implications for the analysis of NCR ligand expression on adherent cells. To avoid artifacts due the detachment of cells by trypsin we used a non-enzymatic cell dissociation reagent in this study.

The increased surveillance of cells in mitosis by human NK cells is blocked by hyaluronidase treatment and antibodies against NKp46 or NKp44 [18]. As hyaluronan and NCR do not seem to interact directly, NCR ligands could be higher expressed in mitotic cells. Surprisingly, we found the opposite by demonstrating a clear down-modulation of NKp30L and NKp44L on cells arrested in mitosis. This suggests that the direct interaction between NCR and their cellular ligands is not involved in the increased surveillance of mitotic cells. Instead NCR might be important for the activation of a hyaluronan-binding receptor.

NK cells are particularly active against cells derived from the hematopoietic system. Interestingly, we found the lowest expression of NKp30L and NKp44L on these cells, consistent with other reports [31], [33]. This could indicate that the NCR may not play a major role in NK cell recognition of hematological disorders. However, ligands for 2B4, CRACC and NTB-A are commonly present on hematopoietic cells [8]. These receptors can effectively co-stimulate NCR-mediated NK cell activation [34]–[36]. This co-stimulation may provide sufficient NCR-mediated NK cell activation even under conditions of low NCR ligand expression. As these co-stimulatory ligands are missing in cell lines derived from solid tumors, a higher expression of NCR ligands might be necessary for efficient NK cell recognition.

## Materials and Methods

### Cells and antibodies

Cell lines used in this study are listed in tables 1 and 2. Primary human NK cells were isolated and cultured as described [37]. Antibodies used were MOPC-21 (Sigma) and phycoerythrine (PE)-conjugated goat anti-mouse IgG (Jackson Immunoresearch, West Grove, PA). Mouse anti-isoleucine-zipper (ILZ-11), mouse anti-NKp30 (p30-15) and mouse anti-NKp44 (p44-8) monoclonal antibodies were created in our laboratory by immunizing BALB/c mice with the respective ILZ fusion protein. Specificity of the resulting hybridoma was confirmed using ELISA and flow cytometry.

### Production of soluble fusion proteins and flow cytometry staining

Isoleucin-zipper fusion proteins were produced as described elsewhere [32]. Ig-fusion proteins were produced similarly [38] but purified using protein A agarose Sepharose beads (Amersham) and eluted with low pH glycin buffer. Resulting proteins were dialyzed against PBS and analyzed for purity on a polyacrylamide gel stained with Safestain (Invitrogen). For flow cytometry, adherent cells were detached using enzyme free cell dissociation buffer (Gibco). 1×10^5^ cells were resuspended in 50 µl staining buffer (PBS/2% FCS) with 1 µg/ml of the respective ILZ fusion protein. After 20 min on ice cells were washed and resuspended in staining buffer with mouse anti-ILZ antibody (ILZ-11, 5 µg/ml) and incubated for 20 min on ice. Then cells were washed again and incubated with PE-conjugated goat anti-mouse antibody (1∶200) for 20 min on ice in the dark. Cells were washed and then fixed with 2% formaldehyde in staining buffer and analyzed on a Becton-Dickinson FACScan flow cytometer using the BD Cellquest and FlowJo software.

### Chromium release assay

Cytotoxicity assays were performed essentially as described [39]. Target cells (0.5×10^6^) were labeled in 100 µl assay medium (IMDM with 10% FCS and 1% penicillin/streptomycin) with 100 µCi (3.7 MBq) of ^51^Cr for 1 h at 37°C. Cells were washed twice and resuspended at 5×10^4^ cells/ml in assay medium. Effector cells were resuspended in assay medium and mixed at different effector to target (E∶T) ratios with 5000 labeled target cells/well in a 96-well V-bottom plate. Where antibodies were included in the assays effector cells were preincubated with the respective antibodies (10 µg/ml final concentration) for 15 min at 37°C before addition of target cells. Maximum release was determined by incubation of target cells in 1% Triton X-100 solution. For spontaneous release, targets were incubated without effectors in assay medium alone. All samples were done in triplicates. Plates were incubated for 4 h at 37°C. Supernatant was harvested and ^51^Cr-release was measured in a γ-counter. Percent specific release was calculated as ([experimental release−spontaneous release]/[maximum release−spontaneous release])×100. The ratio between maximum and spontaneous release was at least 3 in all experiments.

### Trypsin treatment, cell cycle arrest and analysis

For trypsin treatment cells were washed once with PBS and incubated in trypsin solution (0.25 %) for 10 minutes at 37°C. The incubation was stopped by washing the cells once in FCS containing medium and cells were analyzed immediately.

293T cells were seeded in 6-well plates at a density of 0.5×10^6^ cells/well. For cell cycle inhibition cells were treated for 18 hours either with 0.1 µM colchicine or 100 ng/ml nocodazole. DMSO was used as a control. As an additional control cells were treated with 10 µM colchizine for 1 hour directly before fixation. Afterwards, cells were harvested and washed with 1 ml of PBS/0.5% FCS. Then cells were resuspended in 50 µl PBS/0.5% FCS and fixed with 150 µl ice-cold ethanol and stored at −20°C for at least 48 h. Then cells were washed three times using PBS/0.5% FCS and afterwards resuspended in 300 µl propidium iodide staining solution (20 µg/ml PI, 100 µg/ml RNase, 0.1% Triton X-100) and incubated for 30 min at room temperature. Cells were analyzed using a BD FACSCalibur.

### Staining of cytospins

Cytospins were prepared by centrifuging ∼1×10^5^ tumor cells per sample onto glass slides using a Shandon Cytospin 2. Cells were air-dried over night and then fixed in acetone for 10 min at room temperature. Following rehydration in PBS, cell spots were incubated with ∼1 µg purified NKp46-Ig, NKp44-Ig or NKp30-Ig in 50 µl PBS/2% FCS for 45 min at room temperature. After washing of the slides with PBS, cells were incubated with peroxidase-conjugated goat anti-human IgG-Fc (Dianova, Hamburg, Germany) (dilution 1∶200 in PBS/2% FCS) for 30 min at room temperature. The peroxidase was developed using 0.4 mg/ml 3-amino-9-ethylcarbazole (Sigma-Aldrich, Taufkirchen, Germany) in 0.1 M Na acetate buffer (pH 5.2) and 0.015% H_2_O_2_. Monoclonal antibodies recognizing the cellular compartment markers calnexin, GM130, EEA-1 and LAMP-1 were from Becton Dickinson (Heidelberg, Germany). These antibodies were diluted 1∶50 in 50 µl PBS/2% FCS per slide and after binding for 45 min reacted with goat anti-mouse (or rat) IgG-Fc (Dianova, Hamburg, Germany). Slides were mounted in glycerol gelatine (Merck, Darmstadt, Germany). Cells were analyzed using an Olympus BH-2 microscope.
